# Autoimmunity conferred by *chs3-2D* relies on *CSA1*, its adjacent TNL-encoding neighbour

**DOI:** 10.1038/srep08792

**Published:** 2015-03-05

**Authors:** Fang Xu, Chipan Zhu, Volkan Cevik, Kaeli Johnson, Yanan Liu, Kee Sohn, Jonathan D. Jones, Eric B. Holub, Xin Li

**Affiliations:** 1 Michael Smith Laboratories, University of British Columbia, Vancouver, BC V6T 1Z4, Canada; 2 Department of Botany, University of British Columbia, Vancouver, BC V6T 1Z4, Canada; 3 University of Warwick, School of Life Sciences, Warwick Crop Centre, Wellesbourne, CV35 9EF, United Kingdom; 4 Sainsbury Laboratory, Norwich Research Park, Colney Lane, Norwich, NR4 7UH, United Kingdom; 5 Bio-protection Research Centre, Institute of Agriculture and Environment, Massey University, Private Bag 11222, Palmerston North. 4442, New Zealand

## Abstract

Plant innate immunity depends on the function of a large number of intracellular immune receptor proteins, the majority of which are structurally similar to mammalian nucleotide-binding oligomerization domain (NOD)-like receptor (NLR) proteins. *CHILLING SENSITIVE 3* (*CHS3*) encodes an atypical Toll/Interleukin 1 Receptor (TIR)-type NLR protein with an additional Lin-11, Isl-1 and Mec-3 (LIM) domain at its C-terminus. The gain-of-function mutant allele *chs3-2D* exhibits severe dwarfism and constitutively activated defense responses, including enhanced resistance to virulent pathogens, high defence marker gene expression, and salicylic acid accumulation. To search for novel regulators involved in CHS3-mediated immune signaling, we conducted suppressor screens in the *chs3-2D* and *chs3-2D pad4-1* genetic backgrounds. Alleles of *sag101* and *eds1-90* were isolated as complete suppressors of *chs3-2D*, and alleles of *sgt1b* were isolated as partial suppressors of *chs3-2D pad4-1*. These mutants suggest that SAG101, EDS1-90, and SGT1b are all positive regulators of CHS3-mediated defense signaling. Additionally, the TIR-type NLR-encoding *CSA1* locus located genomically adjacent to *CHS3* was found to be fully required for *chs3-2D*-mediated autoimmunity. *CSA1* is located 3.9 kb upstream of *CHS3* and is transcribed in the opposite direction. Altogether, these data illustrate the distinct genetic requirements for CHS3-mediated defense signaling.

Plants have evolved a multi-layered immune system to protect them from various pathogen infections. The first layer of the defense surveillance mechanism relies on the recognition of pathogen-associated molecular patterns (PAMPs) via cell-surface localized pattern-recognition receptors (PRRs)^1^. PAMPs consist of conserved molecular features of pathogens, such as bacterial flagellin and fungal chitin. Recognition by PRRs leads to the induction of PAMP-triggered immunity, which results in mitogen-activated protein kinase cascade activation, generation of reactive oxygen species, callose deposition, and accumulation of the plant defense hormone salicylic acid^1^. Successful pathogens are able to deliver effectors into plant cells to perturb these defense responses, leading to Effector-Triggered Susceptibility. In order to counteract these effectors plants have evolved resistance (R) proteins, which constitute the second layer of the plant immune system. Upon specific recognition of effectors, R protein activation leads to the activation of effector-triggered immunity (ETI). ETI is rapid and robust, often culminating in a hypersensitive response (HR), which is a specific type of programmed cell death^2^.

Most plant R proteins resemble mammalian nucleotide-binding oligomerization domain (NOD)-like receptor (NLR) proteins. Genome-wide analysis reveals that in Arabidopsis there are about 150 typical NLR proteins^3^, which can be further divided into two subgroups based on their N-termini: those with a Toll/Interleukin 1 Receptor (TIR) domain are termed TNLs, and those with a coiled-coil motif are termed CNLs^4^. Through previous genetic studies, it seems that TNLs and CNLs have different requirements for downstream signaling. TNL-mediated signaling relies upon the nucleo-cytoplasmic ENHANCED DISEASE SUSCEPTIBILITY 1 (EDS1)/PHYTOALEXIN DEFICIENT 4 (PAD4)/SENESCENCE-ASSOCIATED GENE101 (SAG101) complex^5^,^6^,^7^. However, CNL signaling likely depends on the membrane-bound NONRACE SPECIFIC DISEASE RESISTANCE 1 (NDR1)^5^,^8^,^9^.

Arabidopsis *CHILLING SENSITIVE 3* (*CHS3*) encodes an atypical TNL protein with a Lin-11, Isl-1 and Mec-3 (LIM) domain at its C terminus^10^. LIM domain-containing proteins are found across eukaryotes and have been implicated as regulators of a variety of biological processes, including but not limited to gene expression and signal transduction^11^. It is hypothesized that the LIM domain may act as a repressor domain in CHS3^10^. Whether this non-canonical TNL employs the same downstream regulators as typical TNLs has yet to be explored.

*chs3-2D* is a gain-of-function mutant isolated from a forward genetic screen designed to isolate defense regulators^12^. In the *chs3-2D* mutant, a C1340 to Y1340 substitution close to the LIM domain of CHS3 leads to autoimmune phenotypes including increased *PATHOGENESIS-RELATED* (*PR*) gene expression, salicylic acid accumulation, and enhanced resistance to the virulent oomycete strain *Hyaloperonospora arabidopsidis* (*H.a.*) Noco2^12^. In this study we carried out two independent suppressor screens in the *chs3-2D* and *chs3-2D pad4-1* genetic backgrounds, respectively, to explore the regulatory and signaling components of CHS3-mediated defense. We report the identification and characterization of multiple mutants that can suppress the *chs3-2D* or *chs3-2D pad4-1* autoimmune phenotypes. Using map-based cloning and Sanger sequencing techniques we were able to clone a number of genes, including novel alleles of known downstream regulators of TNL-mediated signaling, such as SAG101, EDS1, and SUPPESSOR OF THE G2 ALLELE OF SKP1, b (SGT1b)^13^. Most significantly, our study revealed that the autoimmunity of *chs3-2D* requires the genomically adjacent TNL gene *CONSTITUTIVE SHADE-AVOIDANCE 1* (*CSA1*), as four independent mutant alleles of *csa1* were found to suppress the autoimmunity of *chs3-2D*.

## Methods

### Plant growth

Seeds were sterilized by soaking them in a solution of 15% bleach and 0.1% Tween 20 followed by rinsing twice with sterile water. Seeds were cold treated in the dark at 4°C for three days. Plate-grown plants were grown on ½ MS media at 22°C and exposed to a 16 h light and 8 h dark regime.

### Oomycete infection assay

Two-week-old seedlings were spray-inoculated with *H.a.* Noco2 at a spore concentration of 1 × 10^5^ spores per mL. Oomycete growth was scored seven days later. The 16 plants from each genotype were divided into groups of four and placed in 1 mL of ddH_2_O in 15 ml tubes (4 plants per tube). Spores were suspended in solution by vortexing and counted using a hemocytometer. Three independent replicates were performed.

### Gene expression analysis

Total RNA was extracted from 13-day-old seedlings grown on ½ MS media using the RNA Mini-preps Kit (Bio Basic Inc.). Total RNA was then reverse transcribed using Superscript II reverse transcriptase (Applied Biological Materials). The resulting cDNA was used as template for PCR. Both *PR1* and *ACTIN7* were amplified with 28 cycles while *PR2* was amplified with 30 cycles. PCR products were then run on 1% agarose gel containing ethidium bromide and imaged using an AlphaImager HG (AlphaInnotech). The primers used to amplify *ACTIN7* were 5′-CAGAGTCGAGCACAATACCG-3′ and 5′-GGTGTCATGGTTGGTATGGGTC-3′, the primers used to amplify *PR1* were 5′-GTAGGTGCTCTTGTTCTTCCC-3′ and 5′-CACATAATTCCCACGAGGATC-3′, and the primers used to amplify *PR2* were 5′-GCTTCCTTCTTCAACCACACAGC-3′ and 5′-CGTTGATGTACCGGAATCTGAC-3′.

For *CSA1* gene expression analysis, the cDNAs of wild type, *chs3-2D*, *chs3-2D pad4-1*, *chs3-2D eds1-90-10*, and *chs3-2D eds1-90-11* were obtained as described above. Relative *CSA1* expression levels were determined by real-time PCR. The primers used to amplify *CSA1* were 5′-CAAAAACAAGGGAGGTTCTA-3′ and 5′-TTTGGTGCATCCTTGTTATC-3′.

### Map-based cloning and Sanger sequencing

After the secondary screen, *soc* *chs3-2D* and *socp* *chs3-2D pad4-1* mutants in the Col-0 ecotype of *Arabidopsis thaliana* were crossed with the Landsberg *erecta* (L*er*) ecotype. Among the F2 population, the *chs3-2D* locus was genotyped using insertion/deletion marker *MPI7* (5′GTGAATTCCAATTAGACCGCA3′ and 5′TCCTTGATACCGACCGGTGA3′). Plants homozygous for *chs3-2D* were used for further linkage analysis based on plant size and morphology.

## Results

### Identification and characterization of *suppressors of chs3-2D*

The *chs3-2D* autoimmune mutant exhibits severe dwarfism^12^. In order to search for regulatory and signaling components required for CHS3-mediated defense response, we screened for mutants that can suppress the autoimmunity of *chs3-2D*, using suppression of stunted growth as a proxy during the primary screen. *chs3-2D* seeds were first mutagenized by ethyl methanesulfonate (EMS). The M_1_ plants were grown at 28°C to harvest the M2 seeds as *chs3-2D* autoimmunity and concomitant dwarfism are temperature sensitive; the mutant is seedling lethal at 23°C, but fertile at 28°C. The M_2_ population from approximately 2000 M_1_ plants were initially screened for individuals that were significantly larger than the original mutant. Mutants exhibiting a morphological suppression of *chs3-2D*-associated phenotypes were then subjected to a secondary screen, in which resistance to the virulent oomycete strain *H.a.* Noco2 was examined. Mutants that displayed enhanced susceptibility to *H.a.* Noco2 as compared to *chs3-2D* were selected for further characterization. The genetic background of all mutants was verified by directly sequencing the *CHS3* locus. Mutants carrying intragenic mutations in *CHS3* as revealed by the sequencing were classified as intragenic mutants and eliminated from further analysis^12^. In summary, eight independent *soc* (*suppressor of chs3-2D*) lines with second-site mutations were isolated.

**Figure 1:**
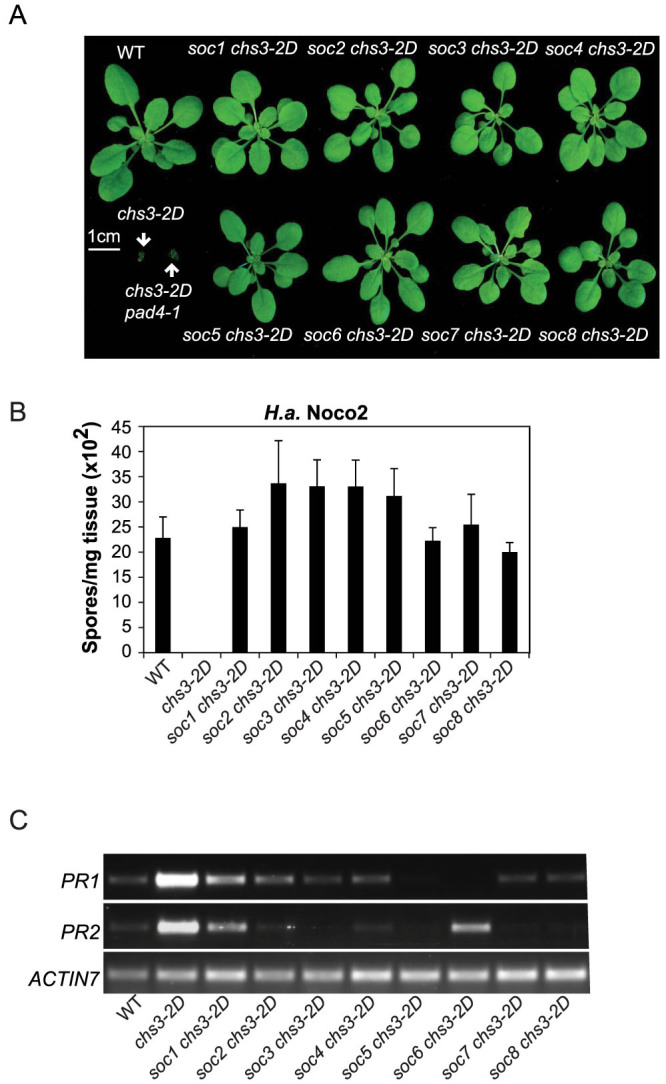
Characterization of *soc* *chs3-2D* mutants. (A) Morphology of soil-grown plants of the indicated genotypes. The photograph was taken when the plants were 21 days old. (B) Quantification of *Hyaloperonospora arabidopsidis* (*H.a.*) sporulation on the indicated genotypes following inoculation with pathogen isolate *H.a.* Noco2. Two-week-old soil-grown seedlings were sprayed with a spore suspension of *H.a.* Noco2 at a concentration of 100,000 spores/mL of water. The plants were then covered and incubated for seven days in a high humidity growth chamber. Spores were counted in water suspension using a hemocytometer (bars represent means of n replicates ± SD, n = 3 or 4 with 4 plants each). (C) *PR1* and *PR2* gene expression of the indicated genotypes as determined by RT-PCR. Both *PR1* and *ACTIN7* were amplified with 28 cycles while *PR2* was amplified with 30 cycles. Two-week-old plate-grown seedlings were used for the analysis.

As shown in Figure 1A, all eight *soc* *chs3-2D* mutants can completely suppress the morphology of *chs3-2D*. Consistent with the morphological suppression, all of them showed significantly enhanced susceptibility to *H.a.* Noco2 (Figure 1B). In addition, the expression of *PR* genes was significantly reduced in all mutants as compared to *chs3-2D* (Figure 1C). Taken together, these data suggest that all of the eight *soc* *chs3-2D* mutants contain mutations that suppress the autoimmune phenotypes of *chs3-2D*.

### *chs3-2D* is only marginally suppressed by *pad4-1*

**Figure 2:**
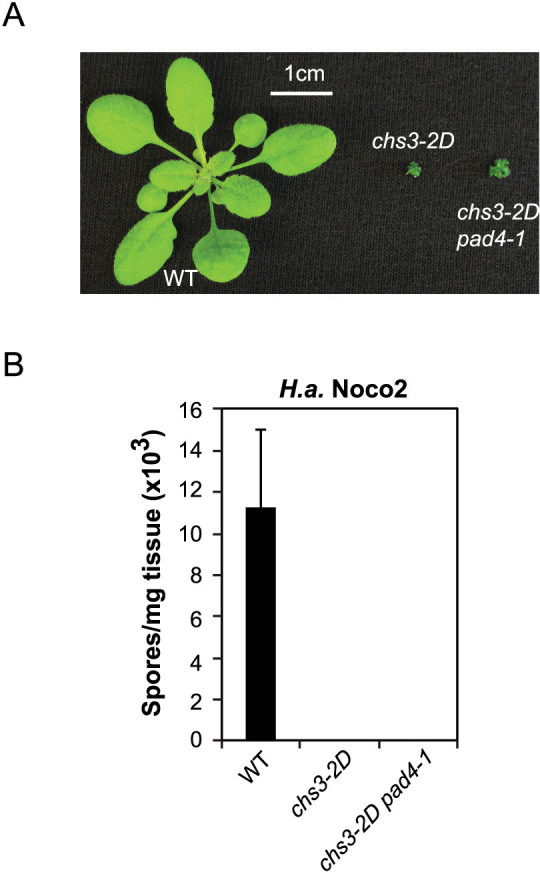
Autoimmunity conferred by *chs3-2D* is only marginally attenuated by *pad4-1*. (A) Morphology of three-week-old soil-grown plants of the noted genotypes. (B) Quantification of *H.a.* Noco2 sporulation on the indicated genotypes. The same experimental procedure was carried out as described in Figure 1B.

In addition to *chs3-2D*, another gain-of-function autoimmune mutant allele, *chs3-1*, was isolated from a forward genetic screen searching for chilling sensitive mutants^10^. Epistasis analysis revealed that the constitutive activation of defense response in *chs3-1* fully depends on EDS1 and partially relies on PAD4, suggesting that a PAD4-independent pathway might play an important function in CHS3-mediated defense response. When we created the *chs3-2D pad4-1* double mutant, the presence of the PAD4-independent pathway in *chs3-2D*-mediated defense response was further confirmed as *pad4-1* only marginally suppressed the *chs3-2D* autoimmune phenotypes. *chs3-2D pad4-1* only showed a slight morphological suppression of the *chs3-2D*-associated dwarfism (Figure 2A) and still exhibited constitutive resistance to *H.a.* Noco2 (Figure 2B).

### Identification and characterization of *suppressors of* *chs3-2D pad4-1*

**Figure 3:**
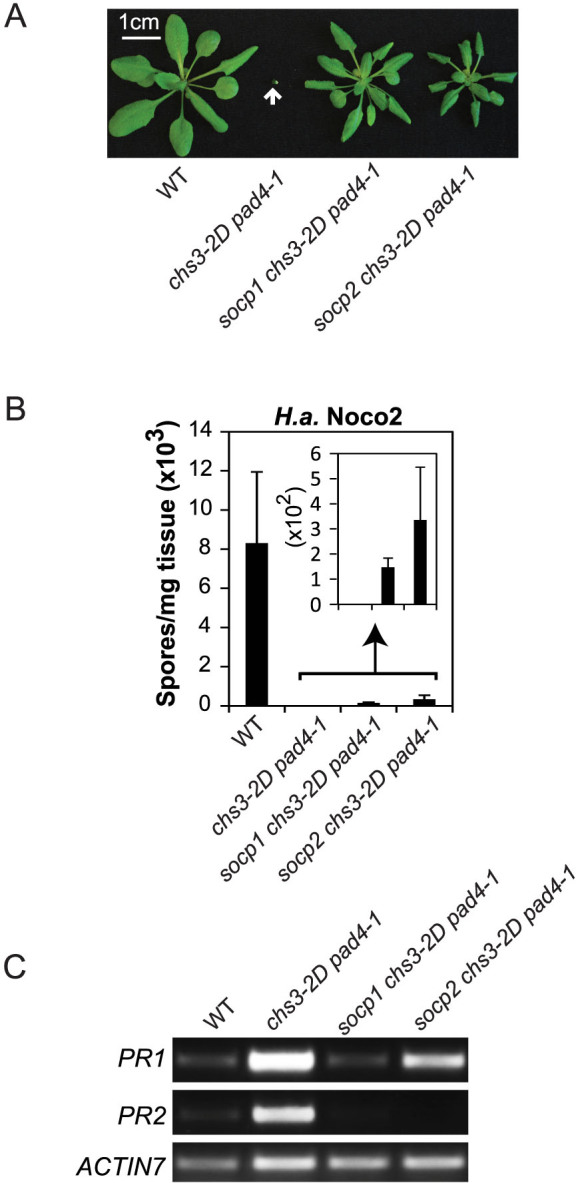
Characterization of *socp chs3-2D pad4-1* mutants. (A) Morphology of soil-grown plants of the indicated genotypes. The picture was taken when the plants were 24 days old. (B) Quantification of *H.a.* Noco2 sporulation on the indicated genotypes. The experimental procedure was carried out as described in Figure 1B. (C) *PR1* and *PR2* gene expression of the indicated genotypes as determined by RT-PCR. Two-week-old plate-grown seedlings were used for the analysis. This image is a cropped version of the original gel picture shown in Supplementary Figure 1.

In order to identify PAD4-independent regulators involved in the *chs3-2D*-mediated signaling pathway, we conducted a suppressor screen in the *chs3-2D pad4-1* background. A similar screening strategy was used as described above for the *chs3-2D* suppressor screen. Two *socp (suppressor of chs3-2D pad4-1)* mutants were isolated from the screen. As shown in Figure 3A, *socp1* *chs3-2D pad4-1* and *socp2* *chs3-2D pad4-1* exhibit significant morphological suppression of *chs3-2D pad4-1*. When the two mutants were challenged with *H.a.* Noco2, they exhibited considerable susceptibility compared to *chs3-2D pad4-1* (Figure 3B). In addition, the constitutive expression of *PR* genes in *chs3-2D pad4-1* was suppressed in the *socp1* *chs3-2D pad4-1* and *socp2* *chs3-2D pad4-1* mutants to some extent (Figure 3C). Therefore, *socp1* and *socp2* were able to partially suppress the autoimmunity of *chs3-2D pad4-1*.

### CHS3-mediated defense responses are completely dependent on EDS1-90 and SAG101

**Figure 4:**
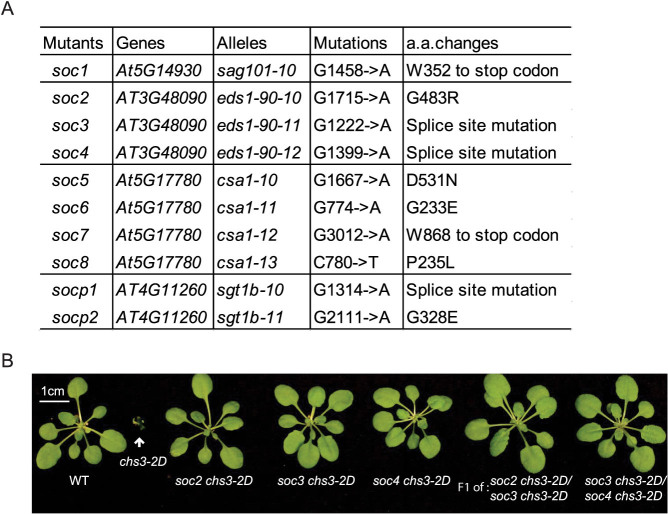
Summary of the mutations found in all the *soc* and *socp* mutants. (A) Mutations identified in the indicated mutants by traditional mapping and Illumina next generation or direct Sanger sequencing. (B) Complementation test of *soc2 chs3-2D, soc3 chs3-2D and soc4 chs3-2D*. Morphology of three-week-old soil-grown plants of the indicated genotypes from the pair-wise allelism test. One representative F1 plant was shown for each cross.

To map the *SOC* loci, the *soc* *chs3-2D* mutants (which were generated in the Columbia (Col-0) ecotype) were crossed with wild type Landsberg *erecta* plants. Crude mapping using insertion/deletion markers specific to the two ecotypes revealed that *soc1* is linked to *SAG101* on chromosome 5. Since SAG101 is a known downstream component of TNL-mediated immunity, we hypothesized that *soc1* might contain a mutation in *SAG101*. Indeed, direct Sanger sequencing revealed that *soc1* carried a G to A mutation in the second exon of *SAG101* (*At5g14930*) resulting in a G1458 to A1458 substitution (Figure 4A). By using similar mapping strategies, we mapped *soc2*, s*oc3* and *soc4* to chromosome 3, close to *EDS1* (*At3g48090*). Sanger sequencing indeed found that all three mutants carried mutations in *EDS1-90*. One *eds1-90* allele had a G to A mutation leading to a G483 to R483 substitution, and the other two alleles contained mutations at intron-exon junctions, which result in splice pattern changes (Figure 4A). Complementation test further confirmed that those three mutants were allelic to each other as they failed to complement each other (Figure 4B).

### PAD4-independent CHS3-mediated defense signaling is partially dependent on SGT1b

Crude mapping of the two *socp* mutants isolated in the *chs3-2D pad4-1* suppressor screen indicated that they both displayed linkage at the top of chromosome 4, a region that contains the known defense regulator *SGT1b*. Sanger sequencing uncovered that *socp1* had a G to A splice site mutation in the fifth intron of *SGT1b*, and *socp2* contained a point mutation leading to a G328 to E328 substitution (Figure 4A). These mutations in *SGT1b* are able to partially suppress the morphological and resistance phenotypes of *chs3-2D pad4-1*, suggesting that SGT1b positively regulates CHS3-mediated defense responses, which might function independently of PAD4.

### Immune signaling mediated by CHS3 requires its neighbour TNL protein CSA1

**Figure 5:**
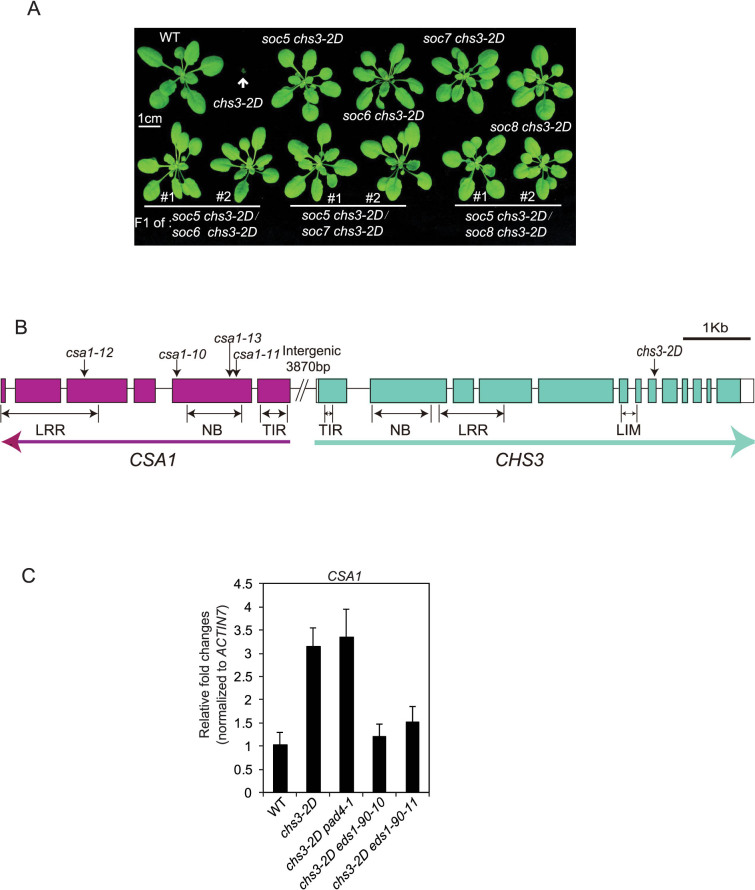
Complementation test of four *csa1* mutations and gene arrangements of *CHS3* and *CSA1*. (A) Morphology of three-week-old soil-grown plants of the indicated genotypes from the pair-wise allelism test. Two representative F_1_ plants were shown for each cross. (B) Boxes indicate exons while lines indicate introns. The encoded protein domains are denoted below the corresponding genomic regions. The directions of gene transcription are indicated as arrows. (C) *CSA1* gene expression in plants of wild type, *chs3-2D*, *chs3-2D pad4-1*, *chs3-2D eds1-90-10*, and *chs3-2D eds1-90-11*. Total RNA was extracted from two-week-old plate-grown seedlings. Relative *CSA1* expression levels were determined by real-time PCR. Values were normalized to the expression of *ACTIN7*. Error bars represent SD from three replicates.

Mapping of *soc5, soc6, soc7* and *soc8* revealed that these suppressor loci were all closely linked with *chs3-2D*. Genomic DNA from *soc6 chs3-2D* was then sequenced using Illumina next generation sequencing. Upon comparison with the Col-0 reference sequence, a point mutation causing a G233 to E233 amino acid substitution was identified in *CSA1*, a gene adjacent to *CHS3*. Direct Sanger sequencing of the remaining *soc* alleles revealed that they contain independent mutations in *CSA1*. This indicates that *CSA1* is required for the autoimmune responses of *chs3-2D*. Complementation was not observed from pair-wise crosses among these mutants, confirming that they contain mutations in the same gene (Figure 5A). *CSA1* is adjacent to and divergently transcribed from *CHS3*, with an approximate 3.9 kb genomic region between their start codons (Figure 5B). It encodes a typical TNL.

When the *CSA1* expression was examined in *chs3-2D* background, we observed over two-fold higher expression of the *TNL* gene in both *chs3-2D* and *chs3-2D pad4-1* (Figure 5C). However, *eds1-90* alleles can completely abolish the up-regulation of *CSA1* in *chs3-2D*, indicating that the induced expression of *CSA1* in *chs3-2D* is mediated through EDS1.

## Discussion

CHS3 is an atypical TNL protein with an additional LIM domain at its C terminus. The exact function of the LIM domain is still unclear. It has been proposed that it inhibits the NLR protein in its native state^10^. The gain-of-function *chs3-2D* allele results in extreme dwarfism and enhanced resistance to virulent pathogens^12^. Epistasis analysis indicates that PAD4, which is thought to be a critical regulator downstream of many TNL immune receptors^14^, is only partially required for the *chs3-2D* phenotypes. The distinctive features of *chs3-2D* provide us with an excellent background in which to conduct genetic suppressor screens in order to identify downstream components involved in CHS3-mediated defense pathway, which seems to differ from those involved in canonical TNL-mediated signaling.

In this study, we determined that CHS3-mediated signaling relies differently on defense-related lipase-like proteins (EDS1/PAD4/SAG101) than signaling pathways downstream of other typical TNLs. Mutations in *SAG101* can completely suppress the autoimmunity of *chs3-2D* (Figure 1 and Figure 4A), while the suppression by *pad4-1* is marginal (Figure 2), suggesting that CHS3-mediated signaling relies more strongly on SAG101. Genetic redundancy between *PAD4* and *SAG101* was previously suggested^6^,^15^. However, previous research provided evidence that EDS1 forms distinct complexes with PAD4 and SAG101 with non-redundant signaling roles^16^. Our findings potentially support this model. Although genetic redundancy between the two Col-0 *EDS1* genes (*EDS1-80* and *EDS1-90*) was demonstrated for immune signaling mediated by the CNL HYPERSENSITIVE RESPONSE TO TCV^17^, CHS3-mediated defense responses seem to rely more on EDS1-90, as three mutations in *EDS1-90*, while none in *EDS1-80*, were found to completely abolish the autoimmunity in *chs3-2D*. It is therefore possible that CHS3 preferentially utilizes EDS1-90 and SAG101 for its defense activation, while EDS1-80 and PAD4 are marginally used.

Previous studies have shown that SGT1b negatively regulates defense responses mediated by the TNL SNC1 (SUPPRESSOR OF NPR1, CONSTITUTIVE 1) by altering its accumulation, as mutations in *SGT1b* lead to higher SNC1 levels^18^,^19^. However, SGT1b appears to positively regulate CHS3-mediated defense responses, as mutations in *SGT1b* can partially suppress the phenotypes of *chs3-2D pad4-1* (Ref. 10 and current study). Together, these data further highlight the differential roles SGT1b plays in NLR-mediated immunity. In the case of CHS3, SGT1b may serve as a molecular chaperon for proper assembly of the TNL complex for defense activation. In contrast, for SNC1, SGT1b is likely more involved in the SCF^CPR1^ complex formation for ubiquitination and further degradation of this TNL^18^.

The results of our suppressor screens have shown that the autoimmunity of *chs3-2D* requires the TNL CSA1, as loss-of-function mutations in *CSA1* can completely suppress the *chs3-2D* phenotypes. *CSA1* is adjacent to and divergently transcribed from *CHS3*, sharing an approximate 3.9 kb genomic region upstream of their start codons. This genomic arrangement is reminiscent of the *R* gene pair *RPS4* and *RRS1*, whose hetero-dimerization is required for effector recognition^20^. They are in a head-in-head arrangement with a 264 bp intergenic region, and are transcribed in opposite directions. The promoter regions probably overlap and this gene pair is likely under transcriptional co-regulation. RRS1, a TNL immune receptor with an extra WRKY domain at the C-terminus, confers recognition of the fungal pathogen *Colletotrichum higginsianum* and effector PopP2 from the bacterial pathogen *Ralstonia solanacearum*, while RPS4 is a typical TNL immune receptor that confers recognition of effector AvrRPS4 from *Pseudomonas syringae* pv. *tomato*^21^,^22^,^23^. Interestingly, both RRS1 and RPS4 are required for resistance conferred by either of the protein pair, revealing a dual resistance gene system^22^,^23^. Structural studies show that TIR domain hetero-dimerization is critical to form a functional RPS4/RRS1 effector interaction interface^20^. It is proposed that upon effector perception, TIR domain hetero-dimerization is released to allow for RPS4 TIR domain homo-dimerization and defense activation^20^. In addition to RPS4/RRS1 and CSA1/CHS3, there are another eight conserved TNL gene pairs in the *Arabidopsis* genome, which suggests the presence of conserved dual resistance gene systems^23^.

Curiously, CSA1 is the closest homolog of RPS4 and over-expression of RPS4 is able to complement the loss-of-function *csa1* phenotype^24^. Single *csa1* mutant plants exhibit constitutive shade avoidance and susceptibility toward avirulent *Pseudomonas* pathogen^24^. Moreover, CHS3 is the closest homolog of RRS1 in the Col-0 ecotype. They have similar domain arrangements, and both the LIM and WRKY domains are proposed to be transcriptional repression domains^25^. In addition, RPS4 is required for the constitutive activation of defense responses conferred by a gain-of-function allele RRS1^SLH1^ which contains a single amino acid insertion in the WRKY DNA-binding domain^26^. Therefore we speculate that CHS3 may function similarly as RRS1. However, the biochemical interaction between CHS3 and CSA1 awaits further examination.

### Proposed working model for CHS3-mediated defense pathway

**Figure 6:**
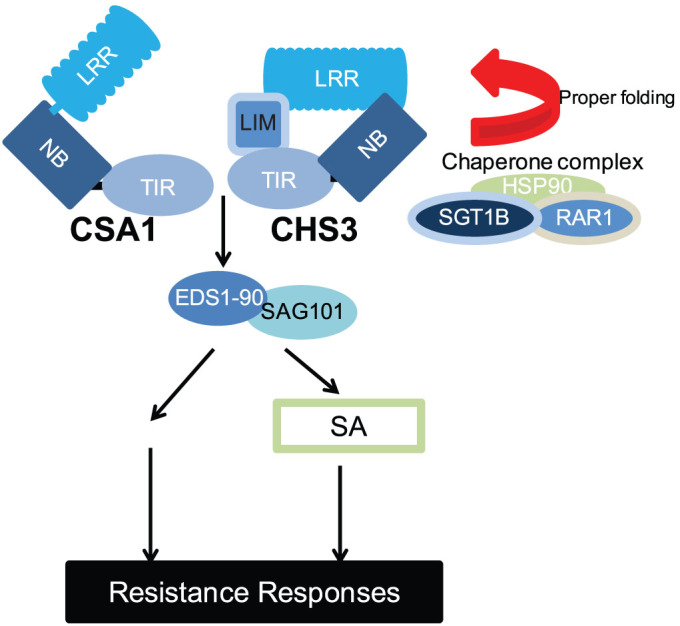
Proposed working model for the CHS3-mediated defense pathway. The LIM domain was proposed to repress CHS3 activities in the absence of pathogens. SGT1b, which can form a chaperone complex with HSP90 and RAR1, is probably required for the proper assembly of CHS3 activation complex. CHS3 activation relies on its TNL neighbour CSA1, and this signaling pathway appears to primarily rely on SAG101 and EDS1-90.

From our suppressor screens, we isolated several downstream regulators of CHS3-mediated signaling. Based on our current genetic data, we propose a working model for the CHS3-mediated pathway. SGT1 may form a chaperone complex with RAR1 and HSP90 to properly assemble the CHS3 activation complex. The C-terminal LIM domain has been hypothesized to inhibit CHS3 protein activation in the absence of pathogens. Moreover, CHS3 needs its TNL protein neighbour, CSA1 to confer defense responses. The interaction between these two TNL proteins *in planta* will be investigated in the future. Instead of relying on the EDS1/PAD4/SAG101 module, CHS3 signaling seems to preferentially employ the EDS1-90/SAG101 complex (Figure 6).

## Author Contributions

F.X., C.Z. and X.L. designed the experiments and wrote the manuscript; F.X. and C.Z. performed all the experiments described in the figures; K.J. and Y.L. performed primary screens. V.C., J.D.J. and E.B.H. contributed to the cloning of *CSAI*. All authors reviewed the manuscript.

## Supplementary Material

Supplementary Information Supplementary Figure 1
