# Supplementary material for: Autoimmunity conferred by chs3-2D relies on CSA1, its adjacent TNL-encoding neighbour
Source: Sci Rep. 2015 Mar 5;5:8792. doi: 10.1038/srep08792 (PMC4350097; doi:10.1038/srep08792)
Supplement: Supplementary Information — Supplementary Figure 1 [file srep08792-s1.pdf]

Supplementary information:

Autoimmunity conferred by *chs3-2D* relies on *CSA1*, its adjacent TIR-NB-LRR-encoding neighbour

Fang Xu, Chipan Zhu, Volkan Cevik, Kaeli Johnson, Yanan Liu, Kee Sohn, Jonathan D. Jones, Eric B. Holub and Xin Li

Supplementary Figure 1. ***PR1 and PR2*** **expression in plants of the indicated genotypes as determined by RT-PCR.**

PR1 and *ACTIN7* were amplified with 28 cycles, and *PR2* was amplified with 30 cycles of PCR. The last 3 lanes plus the WT control panel were cropped and merged into Figure 3C. The gels were run under the same conditions and were processed in parallel.

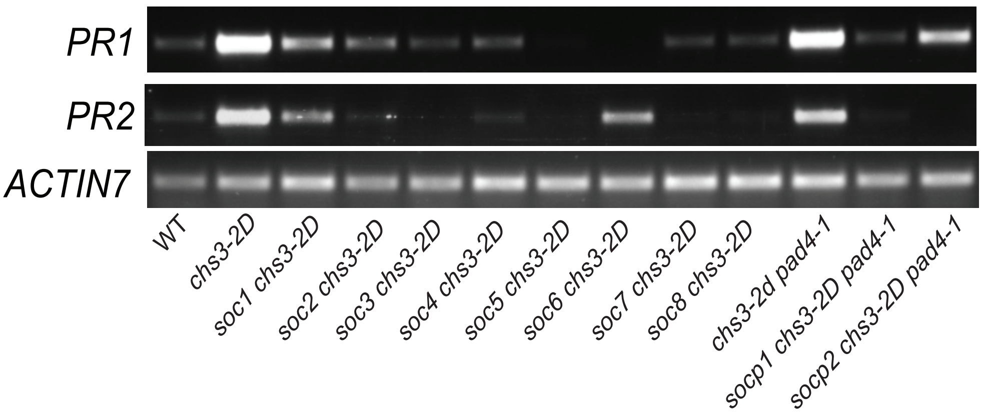
PR1
PR2
ACTIN7
WT
chs3-2D
soc1 chs3-2D
soc2 chs3-2D
soc3 chs3-2D
soc4 chs3-2D
soc5 chs3-2D
soc6 chs3-2D
soc7 chs3-2D
soc8 chs3-2D
chs3-2d pad4-1
socp1 chs3-2D pad4-1
socp2 chs3-2D pad4-1
